# Prospective analysis of metabolic syndrome and inflammation in aortic aneurysm risk: UK Biobank study

**DOI:** 10.3389/fendo.2025.1612975

**Published:** 2025-07-04

**Authors:** Xinyi Liu, Hao Liu, Suwei Chen, Chen Gong, Yipeng Ge, Zhiyu Qiao, Chengnan Li, Junming Zhu

**Affiliations:** ^1^ Department of Cardiovascular Surgery, Beijing Aortic Disease Center, Beijing Anzhen Hospital of Capital Medical University, Beijing, China; ^2^ Department of Hematology, Beijing Anzhen Hospital of Capital Medical University, Beijing, China

**Keywords:** metabolic syndrome, INFLA scores, chronic inflammation, aortic aneurysm, UK Biobank

## Abstract

**Context/Objectives:**

Metabolic syndrome (MetS) is associated with various diseases, yet its connection with aortic aneurysm (AA) is not well understood. The role of chronic inflammation as a mediator in this relationship also remains unclear. This study explores the combined effects of MetS and inflammation on AA risk.

**Methods:**

Data from 312,505 UK Biobank participants were analyzed to assess the relationship between MetS and AA. Cox proportional hazards regression models evaluated the association, while restricted cubic splines, mediation analyses, interaction assessments, and joint analyses explored the impact of inflammatory indicators, including the low-grade chronic inflammation (INFLA) score.

**Results:**

Over a mean follow-up of 14.6 years, 2,382 participants developed AA. MetS was associated with a higher AA risk (HR: 1.27; 95% CI: 1.16–1.39) in fully adjusted models. Each additional MetS component increased AA risk by 16%. Inflammatory markers, including the INFLA score, significantly mediated this relationship. Joint analyses revealed a stronger association in MetS patients with high INFLA scores (HR: 1.68; 95% CI: 1.45–1.95).

**Conclusion:**

MetS and its components notably elevate AA risk, with inflammation playing a key mediating role. These findings underscore the importance of targeted prevention strategies, particularly for MetS populations with high chronic inflammation.

## 1 Introduction

Aortic aneurysm (AA) is a vascular disease characterized by irreversible dilation of the aorta (1). AA typically progresses insidiously, with most patients remaining asymptomatic until severe complications, such as rupture or dissection, occur—events associated with mortality rates exceeding 60% (2). While open surgery and endovascular aortic repair are treatment options, they are limited to advanced cases, and no effective pharmacological interventions exist for early-stage AA (3). Therefore, understanding the factors influencing AA development is critical to devising effective prevention strategies and mitigating its potentially catastrophic outcomes.

Metabolic syndrome (MetS) comprises a cluster of metabolic abnormalities, including central obesity, hypertension, hyperglycemia, hypertriglyceridemia, and dyslipidemia. Affecting 20–30% of adults globally (4), its prevalence continues to rise, particularly among young individuals and women. As a significant public health concern, MetS is strongly associated with an elevated risk of cardiovascular disease, cancer, and other health conditions (5, 6). However, limited evidence exists regarding the potential association between MetS and the development of AA, warranting further investigation.

Chronic inflammation indicated by C-reactive protein (CRP) and white blood cell (WBC) count, has been reported to be closely associated with both MetS (7) and AA (8). Among these, the low-grade chronic inflammation (INFLA) score, representing systemic low-grade inflammation, has been extensively studied in various diseases (9, 10). Despite this, the potential mediating role of chronic inflammation in the relationship between MetS and AA remains unclear.

This study aims to investigate the association between MetS, its individual components, and the risk of developing AA using prospective data from the UK Biobank. Furthermore, it explores the potential mediating role of chronic inflammatory indicators and examines the combined effects of MetS and inflammation on the risk of AA.

## 2 Methods

### 2.1 Study design and participants

We utilized data from the UK Biobank study (Application Number 145937), a large-scale prospective cohort that enrolled over 500,000 participants aged 37 to 73 years from 22 assessment centers across the United Kingdom between 2006 and 2010. Participants provided extensive health-related information through a touchscreen questionnaire, covering demographics, socio-economic status, lifestyle factors, and health conditions. The study’s design and data collection procedures have been thoroughly detailed in prior publications (11).

The cohort initially comprised 370,880 participants with complete data on MetS components at baseline. Following the exclusion of individuals with a history of AA or connective tissue disease at baseline, as well as those with missing data on the INFLA component or other covariates, the final analysis included 312,505 participants (**Supplementary Figure 1**).

### 2.2 MetS assessment

MetS was defined according to the International Diabetes Federation (IDF) criteria as the presence of three or more of the following components: central obesity, hypertension, hyperglycemia, hypertriglyceridemia, and dyslipidemia characterized by low levels of high-density lipoprotein cholesterol (HDL-C) (12). Central obesity was determined using waist circumference thresholds of ≥88 cm for women and ≥102 cm for men. Blood pressure was assessed using two measurements of systolic blood pressure (SBP) and diastolic blood pressure (DBP), with the average values calculated. Hypertension was defined as SBP ≥ 130 mmHg, DBP ≥ 85 mmHg, or a history of antihypertensive medication use. For hyperglycemia, glycated hemoglobin (HbA1c) was used as a more stable metric due to limited fasting glucose measurements among participants. Impaired glucose regulation was identified as HbA1c ≥ 42.0 mmol/mol or a history of glucose-lowering medication. Hypertriglyceridemia was defined as triglyceride levels ≥ 1.70 mmol/L or a history of triglyceride-lowering medication. Reduced HDL-C levels were defined as <1.03 mmol/L for men and <1.29 mmol/L for women, or a history of cholesterol-lowering medication use.

### 2.3 Assessment of inflammation index

To explore the role of inflammation in the association between MetS and AA incidence, we analyzed various inflammatory markers, including WBC count, platelet count, CRP levels, neutrophil count, lymphocyte count, and the neutrophil-to-lymphocyte ratio (NLR). Additionally, we calculated the INFLA score as a comprehensive measure of individual inflammatory status. Based on prior research, the INFLA score, strongly associated with multiple diseases (13, 14), integrates four inflammatory indicators: CRP, WBC count, platelet count, and NLR.

To calculate the INFLA score, each inflammatory marker was natural log-transformed. Biomarker levels within the highest decile (7th to 10th) were assigned scores ranging from +1 to +4, while those within the lowest decile (1st to 4th) were assigned scores ranging from −4 to −1.The resulting INFLA score ranged from -16 to +16, with higher scores reflecting elevated levels of low-grade inflammation (15). As part of a sensitivity analysis, we also developed a weighted INFLA score, assigning weights to each inflammatory marker according to its relative association with the outcome event.

### 2.4 Assessment of covariates

Covariates encompassed demographics, baseline medical history, and lifestyle factors. Demographic variables included age, sex, education level, self-reported race, employment status, and body mass index (BMI). Baseline medical history covered the presence of chronic respiratory disease, chronic kidney disease, chronic liver disease, and cardiovascular disease.

Lifestyle factors were assessed based on six components aligned with World Health Organization guidelines: dietary habits, sleep patterns (categorized as healthy, moderate, or unhealthy), physical activity levels (high, moderate, or low), sedentary behavior (low, moderate, or high), and smoking and alcohol consumption history. Detailed information on the lifestyle assessments is provided in (**Supplementary Tables 1**, **2**).

### 2.5 Outcomes

The primary outcome for this study was AA (International Classification of Diseases, 10th Revision [ICD-10] I71.1–I71.9). Cases of AA were ascertained through death registries, primary care records, hospital admission data, and self-reported diagnoses. The date of AA onset was defined as the date of the first reported diagnosis. Follow-up duration was calculated from the baseline assessment (2006–2010) to the earliest occurrence of AA diagnosis, death, loss to follow-up, or the end of the follow-up period, whichever occurred first.

### 2.6 Statistical analysis

Baseline characteristics of participants were summarized as means with standard deviations (SD) for continuous variables and as proportions for categorical variables. Statistical comparisons for continuous variables were conducted using analysis of variance (ANOVA), while the χ² test was employed to evaluate differences in categorical variables.

Kaplan-Meier curves were generated to estimate the cumulative incidence of AA in relation to MetS and its components. Cox proportional hazards regression models were applied to calculate hazard ratios (HRs) and 95% confidence intervals (CIs) for the risk of AA associated with MetS, its individual components, and the INFLA score. Three multivariable adjustment models were developed. Model 1 adjusted for age and gender. Model 2 included additional adjustments for education, self-reported ethnicity, the Thomson Deprivation Index, employment status, BMI, and medical comorbidities such as a history of cardiovascular disease, chronic respiratory disease, chronic kidney disease, or chronic liver disease. Model 3 further incorporated adjustments for personal lifestyle factors, including diet, sleep patterns, sedentary behavior, physical activity levels, and smoking and alcohol consumption.

The two-stage regression method for survival data, as proposed by VanderWeele (16), was applied to perform mediation analysis, assessing the relationship between MetS and its components (exposures) and AA (outcome) mediated through the INFLA score and its components (mediators). To enhance the stability and reliability of the results, a resampling procedure with 500 iterations was utilized. After adjusting for covariates in Model 3, restricted cubic splines (RCS) were employed to investigate the relationships between MetS components, the INFLA score, and its individual components with the risk of AA.

Stratified analyses were conducted based on MetS status to examine the associations between MetS, INFLA scores, and the development of AA. Participants with low INFLA scores were used as the reference group. Both additive and multiplicative interactions were quantified in this analysis. HRs with 95% CIs were calculated to evaluate interactions on the multiplicative scale, while relative excess risk due to interaction (RERI) with corresponding 95% CIs was used to assess interactions on the additive scale.

To evaluate the joint association, participants were divided into six groups based on the combination of MetS presence and INFLA scores. The reference group included individuals without MetS and with lower INFLA scores. HRs for AA occurrence were calculated for each group. Additionally, subgroup analyses were performed to examine potential differences, stratifying participants by sex (male and female), age (≥60 years and <60 years), BMI (normal BMI: 18.5–24.9 and abnormal BMI), smoking status, and the presence of hypertension. Interactions between these subgroups and the potential mediating effect of INFLA were also analyzed.

In this study, several sensitivity analyses were conducted to evaluate the robustness and consistency of the models. First, adjustments were made for individual inflammatory markers instead of the INFLA score. Second, weighted INFLA scores were constructed to explore the associations between specific inflammatory markers and outcomes. Third, individuals with other medical conditions (cardiovascular disease, chronic liver disease, chronic kidney disease, chronic respiratory disease) were excluded to examine the influence of comorbidities on the results. Fourth, events occurring within the first three years of follow-up were excluded to mitigate potential bias from early events. Fifth, multiple imputation was used to address missing covariate data and assess the impact of incomplete variables on the findings. Finally, to ensure comparability between participants with differing confounding factors, 1:1 propensity score matching (PSM) was performed based on demographic characteristics, lifestyle factors, and comorbidities. A stringent caliper width of 0.1 standard deviations of the propensity score was applied to optimize matching precision.

All statistical analyses were conducted using R software version 4.4.1 (R Foundation for Statistical Computing). P-values were two-sided, and a threshold of P < 0.05 was used to determine statistical significance.

## 3 Results

### 3.1 Baseline characteristics of participants

The baseline characteristics of the participants are presented in **Table 1**. The study included 312,505 individuals with a mean (SD) age of 56.4 (8.1) years. Of these, 147,087 (47.1%) were male, 299,065 (95.7%) were white, and 90,362 (28.9%) had MetS. Participants with MetS, compared to those without, were more likely to be male, have lower levels of education, adopt less healthy lifestyles, and report a greater prevalence of other medical conditions.

**Table 1:** Study participants’ baseline characteristics based on metabolic syndrome status.

Characteristics	Nonmetabolic syndrome (n=222143)	Metabolic syndrome (n=90362)	P value
Demographics			
Age (years)	55.6 ± 8.13	58.5 ± 7.56	<0.001
Male (%)	97420 (43.9%)	49667 (55.0%)	<0.001
White ethnicity or race (%)	213140 (95.9%)	85925 (95.1%)	<0.001
Townsend deprivation index	-1.61 ± 2.89	-1.28 ± 3.05	<0.001
University or college degree (%)	84198 (37.9%)	24487 (27.1%)	<0.001
Employed, student, or retired (%)	203882 (91.8%)	81740 (90.5%)	<0.001
BMI	25.8 ± 3.60	30.8 ± 4.60	<0.001
Lifestyle			
Physical activity (%)			<0.001
Low	64751 (29.1%)	36919 (40.9%)	
Moderate	74832 (33.7%)	29718 (32.9%)	
High	82560 (37.2%)	23725 (26.3%)	
Sleep patterns (%)			<0.001
Poor	81102 (36.5%)	24682 (27.3%)	
Moderate	131723 (59.3%)	58675 (64.9%)	
Good	9318 (4.19%)	7005 (7.75%)	
No heavy alcohol (%)	101204 (45.6%)	49691 (55.0%)	<0.001
Never smoking (%)	127800 (57.5%)	43980 (48.7%)	<0.001
Healthy diet (%)	21251 (9.57%)	8680 (9.61%)	0.739
Sedentary time (%)			<0.001
High	39164 (17.6%)	24029 (26.6%)	
Moderate	71995 (32.4%)	33264 (36.8%)	
Low	110984 (50.0%)	33069 (36.6%)	
Medical history			
Chronic respiratory diseases (%)	27839 (12.5%)	13185 (14.6%)	<0.001
Chronic liver disease (%)	516 (0.23%)	533 (0.59%)	<0.001
Chronic kidney disease (%)	473 (0.21%)	553 (0.61%)	<0.001
Cardiovascular disease (%)	16676 (7.51%)	16242 (18.0%)	<0.001
Metabolic syndrome related components			
Hypertension (%)	135493 (61.0%)	85475 (94.6%)	<0.001
Hyperglycemia (%)	2780 (1.25%)	21344 (23.6%)	<0.001
Dyslipidemia (%)	31002 (14.0%)	66421 (73.5%)	<0.001
Hypertriglyceridemia (%)	51605 (23.2%)	72855 (80.6%)	<0.001
Central obesity (%)	32997 (14.9%)	67092 (74.2%)	<0.001
Inflammation			
White blood cell count	6.62 ± 1.91	7.38 ± 2.11	<0.001
Platelet count	252 ± 57.9	253 ± 61.7	<0.001
Lymphocyte count	1.90 ± 1.06	2.11 ± 1.23	<0.001
Neutrophil count	4.06 ± 1.33	4.51 ± 1.42	<0.001
C-reactive protein	2.07 ± 3.84	3.37 ± 4.50	<0.001
NLR	2.33 ± 1.15	2.35 ± 1.36	<0.001
INFLA score	2.28 ± 5.61	4.77 ± 5.42	<0.001

P values were determined using the ANOVA test for continuous variables and the chi-square test for categorical variables. BMI, Body mass index; NLR, Neutrophil-to-Lymphocyte Ratio; INFLA score, Low-grade chronic inflammation score.

### 3.2 Association of MetS and inflammation with AA incidence

**Figure 1:**
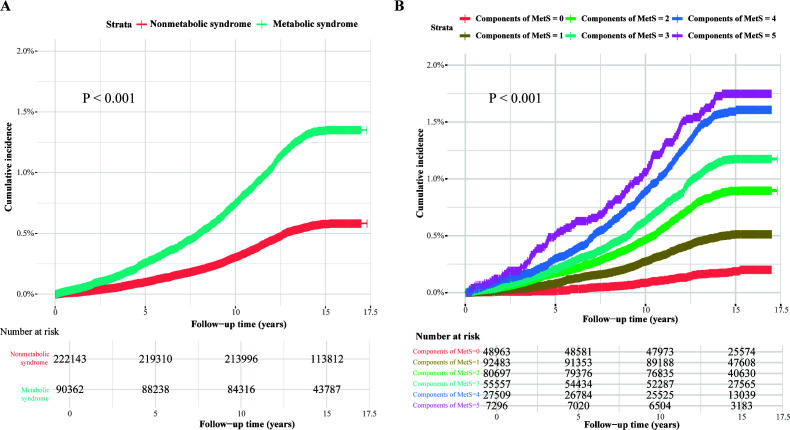
Kaplan-Meier curves of the cumulative incidence of AA caused by MetS and its components. Kaplan-Meier curves of the cumulative incidence of AA. **(A)** The presence or absence of MetS is used as the reference group. **(B)** The individual components of MetS are used as the reference group.

As illustrated by the Kaplan-Meier curves in **Figure 1**, MetS and its individual components were significantly associated with the cumulative incidence of AA. Moreover, a dose-dependent relationship was evident, with the cumulative incidence of AA increasing markedly as the number of MetS components rose.

During a mean follow-up period of 14.6 years, 2,382 cases of AA were identified among the 312,505 participants. The associations between MetS, its components, the INFLA score, and AA incidence are summarized in **Table 2**; **Supplementary Table 3**. In the fully adjusted model, which accounted for the INFLA score and other covariates, MetS was significantly associated with an increased risk of AA (HR: 1.27; 95% CI: 1.16–1.39). When analyzed as a linear variable, each additional MetS component was associated with a 16% higher risk of AA (HR: 1.16; 95% CI: 1.11–1.21). Among the five MetS components, hypertension (HR: 1.25; 95% CI: 1.10–1.43), central obesity (HR: 1.29; 95% CI: 1.16–1.45), hypertriglyceridemia (HR: 1.13; 95% CI: 1.04–1.23), and dyslipidemia (HR: 1.38; 95% CI: 1.26–1.51) were significantly associated with an increased risk of AA. However, hyperglycemia (HR: 0.93; 95% CI: 0.82–1.06) did not demonstrate a significant association after adjusting for all covariates. The results demonstrated robust consistency across all sensitivity analyses, including those incorporating weighted INFLA scores and excluding events occurring within the first three years of follow-up. The propensity score-matched dataset characteristics are presented in **Supplementary Figure 2**. Notably, the primary findings remained consistent even after propensity score matching, as shown in **Supplementary Table 4**.

**Table 2:** Association of aortic aneurysm with metabolic syndrome and INFLA score.

Characteristics	Unadjusted for INFLA score		Adjusted for INFLA score	
	HR (95% CI)	P value	HR (95% CI)	P value
Presence of MetS				
Model 1	1.65 (1.52-1.79)	<0.001	1.56 (1.44-1.69)	<0.001
Model 2	1.36 (1.23-1.49)	<0.001	1.32 (1.20-1.45)	<0.001
Model 3	1.29 (1.18-1.42)	<0.001	1.27 (1.16-1.39)	<0.001
Per component increment				
Model 1	1.28 (1.24-1.32)	<0.001	1.25 (1.21-1.29)	<0.001
Model 2	1.19 (1.15-1.25)	<0.001	1.18 (1.13-1.23)	<0.001
Model 3	1.17 (1.12-1.22)	<0.001	1.16 (1.11-1.21)	<0.001
Hypertension				
Model 1	1.49 (1.32-1.69)	<0.001	1.42 (1.25-1.61)	<0.001
Model 2	1.27 (1.12-1.45)	<0.001	1.24 (1.09-1.41)	<0.001
Model 3	1.28 (1.12-1.46)	<0.001	1.25 (1.10-1.43)	<0.001
Central obesity				
Model 1	1.51 (1.39-1.64)	<0.001	1.43 (1.31-1.54)	<0.001
Model 2	1.38 (1.23-1.54)	<0.001	1.35 (1.21-1.52)	<0.001
Model 3	1.31 (1.17-1.47)	<0.001	1.29 (1.16-1.45)	<0.001
Hypertriglyceridemia				
Model 1	1.25 (1.15-1.36)	<0.001	1.19 (1.09-1.29)	<0.001
Model 2	1.19 (1.09-1.29)	<0.001	1.16 (1.07-1.26)	<0.001
Model 3	1.15 (1.06-1.25)	<0.001	1.13 (1.04-1.23)	0.005
Dyslipidemia				
Model 1	1.88 (1.73-2.04)	<0.001	1.83 (1.68-1.98)	<0.001
Model 2	1.43 (1.31-1.56)	<0.001	1.41 (1.29-1.54)	<0.001
Model 3	1.38 (1.27-1.51)	<0.001	1.38 (1.26-1.51)	<0.001
Hyperglycemia				
Model 1	1.23 (1.09-1.39)	<0.001	1.17 (1.04-1.32)	<0.01
Model 2	0.98 (0.87-1.11)	0.772	0.96 (0.85-1.09)	0.526
Model 3	0.95 (0.83-1.07)	0.379	0.93 (0.82-1.06)	0.261

Model 1, age and gender only; Model 2, further adjusting for education, self-reported ethnicity, Thomson deprivation index, employment, body mass index, and prevalent comorbidities (including history of cardiovascular diseases, chronic respiratory disease, chronic kidney disease, or chronic liver disease); Mode 3, with further adjustments to personal lifestyle, including diet, sleep patterns, sedentary time, physical activity, and smoking and alcohol consumption. INFLA score, Low-grade chronic.

The mediating effect of inflammation-related markers on the association between MetS and AA is presented in **Supplementary Figure 3**. CRP, WBC, platelet count, lymphocyte count, neutrophil count, and the INFLA score were all identified as significant mediators. The respective mediating proportions were 3.9%, 11.4%, 1.7%, 2.8%, 8.9%, and 6.7%. Additionally, the mediating effect of the INFLA score on the relationship between individual MetS components and AA is detailed in **Supplementary Figure 4**. Notably, the INFLA score significantly mediated the associations between central obesity (3.9%), hypertriglyceridemia (13.1%), dyslipidemia (1.9%), and hypertension (8.6%) with AA occurrence, while no significant mediating effect was observed for hyperglycemia.

### 3.3 Interaction and joint analysis of MetS and INFLA scores with AA incidence

**Figure 2:**
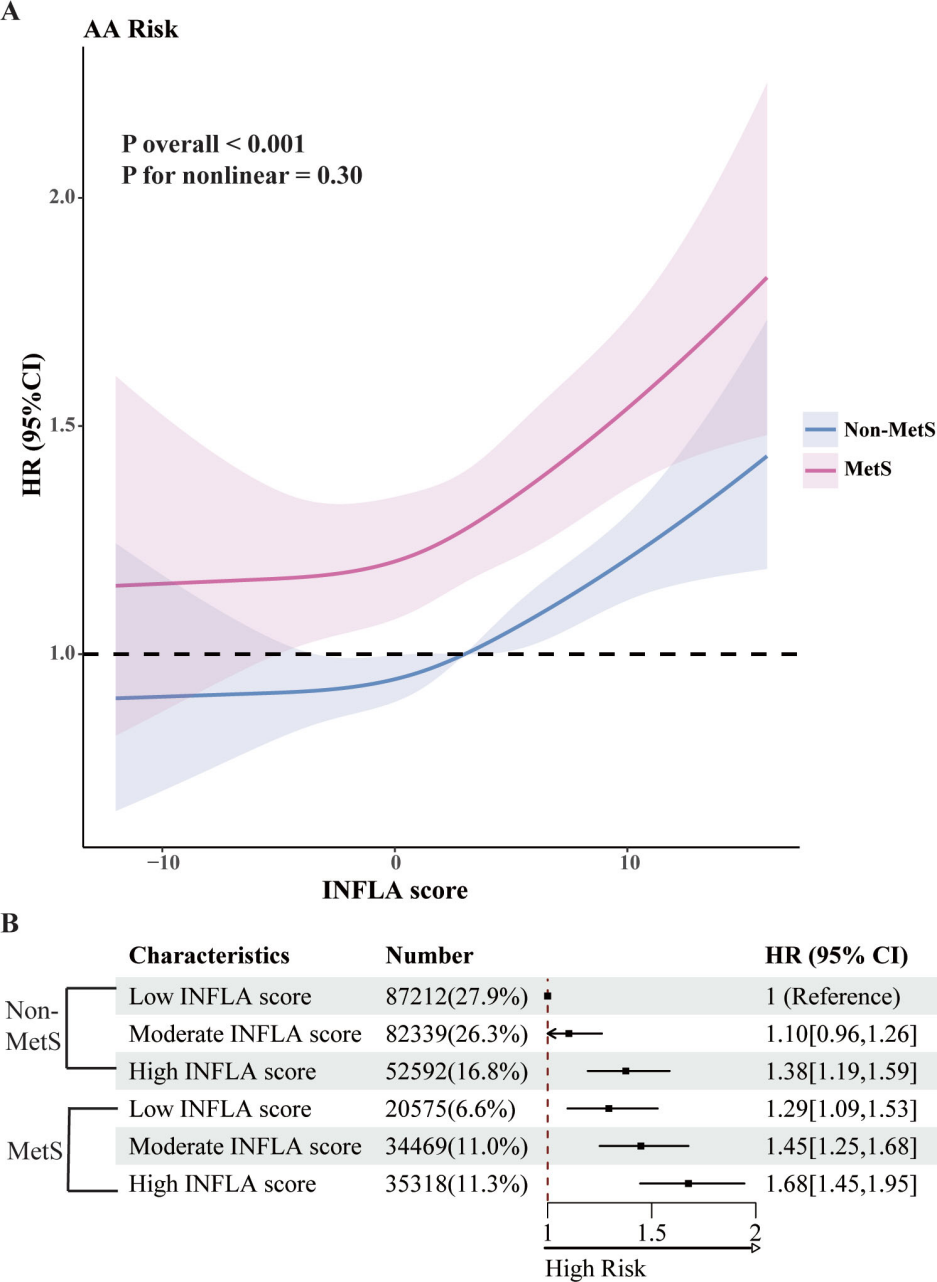
Joint analysis of MetS and INFLA scores. **(A)** The nonlinear relationships between MetS and INFLA scores and AA incidence were observed. **(B)** Joint Cox regression analysis of MetS and INFLA scores. All analyses were adjusted for age, sex, education, self-reported ethnicity, Thomson deprivation index, employment, body mass index, and prevalent comorbidities (including history of cardiovascular disease, chronic respiratory disease, chronic kidney disease, or chronic liver disease) and lifestyle (including diet, physical activity, sleep patterns, sedentary time, and smoking and alcohol consumption).

We used restricted cubic splines to illustrate the dose-response relationships between MetS-related metabolic markers and the risk of AA. As shown in **Supplementary Figure 5**, significant nonlinear associations were observed for diastolic blood pressure, HbA1c, triglycerides, and HDL-C with AA risk. Specifically, the risk of AA increased substantially with higher diastolic blood pressure and triglyceride levels. Conversely, higher levels of HbA1c and HDL-C were associated with a reduced AA risk, with the protective effect of HDL-C plateauing at approximately 2 mmol/L. Additionally, we analyzed the dose-response relationships between inflammatory markers, MetS, and AA risk (**Supplementary Figure 6**). Significant nonlinear associations were observed for CRP, WBC, and platelet counts, with AA risk increasing markedly as levels of these inflammatory indicators rose. Finally, we evaluated the relationship between the INFLA score and AA incidence (**Figure 2A**). A linear association was observed, indicating that the risk of AA increased significantly with higher INFLA scores. Compared with individuals without MetS, those with MetS demonstrated a consistently higher risk of AA across all levels of the INFLA score.

The joint association of MetS and INFLA scores with AA incidence is illustrated in **Figure 2B**. Compared to non-MetS participants with low INFLA scores, individuals with both MetS and high INFLA scores demonstrated a significantly elevated HR of 1.68 (95% CI: 1.45–1.95).

Within the MetS population, participants with high INFLA scores exhibited an approximately 27% increased risk of developing AA compared to those with low INFLA scores (HR: 1.27; 95% CI: 1.08–1.49), as shown in **Supplementary Figure 7**. However, no significant interactions were observed between INFLA scores and MetS on either the multiplicative or additive scale concerning AA incidence (P > 0.05).

### 3.4 Subgroup analysis

The results of the subgroup analysis stratified by sex, age, BMI, smoking status, and the presence of hypertension are presented in **Supplementary Table 5**. The mediating effect of the INFLA score on the association between MetS and AA was significant across all subgroups. The mediating proportion was highest in women (approximately 9.9%) and lowest in non-smokers (approximately 3.3%). Despite these variations in mediating effects, no significant interactions were identified within any of the subgroups.

## 4 Discussion

This study is the first to systematically examine the association between MetS and AA in a large cohort. Our findings reveal that MetS significantly increases the risk of AA, with dyslipidemia contributing the highest risk among the MetS components. Moreover, the INFLA score and its constituent inflammatory markers were found to partially mediate the effect of MetS on AA risk. Notably, MetS patients with high INFLA scores exhibited the highest risk of developing AA, underscoring the critical role of systemic inflammation in the context of metabolic dysfunction.

Our findings add to the growing body of evidence linking MetS and its components to the risk of aortic aneurysm. These results align with the study by Zhao et al. conducted in a Korean population (17), which also demonstrated that MetS and its components significantly increase the risk of aortic aneurysm. Furthermore, multiple components of MetS are strongly associated with the development of aortic aneurysm. The well-established link between hypertension and aortic aneurysm has been confirmed in numerous studies (18–20). Koba et al. demonstrated that elevated triglyceride levels and reduced HDL-C significantly increase the risk of AA in a Japanese cohort (21). Similarly, Sheng et al. reported a significant positive correlation between central obesity and the risk of aortic aneurysm (22). Our study adds further evidence to support the association between these MetS components and AA.

Interestingly, although numerous studies have identified hyperglycemia as a protective factor against AA (23, 24), our findings suggest a more nuanced relationship. After adjusting for all covariates, we observed a significant inverse association between HbA1c levels and AA risk. However, no significant relationship was detected between hyperglycemia and AA. This discrepancy may stem from the relatively small proportion of participants in our cohort with fasting glucose data, potentially omitting a subset of individuals with hyperglycemia. Further follow-up studies with larger and more comprehensive datasets are necessary to better understand the relationship between hyperglycemia and AA.

Currently, limited studies have investigated the pathophysiological mechanisms underlying the role of MetS in AA development. Using transcriptomic data from MetS and AA, Wang et al. suggested that MetS may drive AA progression through the activation of inflammatory pathways, oxidative stress, or extracellular matrix degradation (25). These findings align with our study, which demonstrated a significant mediating role of inflammation in the association between MetS and AA. However, the precise molecular mechanisms involved in the interplay between MetS and AA remain unclear, warranting further research to elucidate these interactions.

Previous studies have established a strong link between chronic inflammation, MetS (26), and AA (27). In this study, we were the first to propose the mediating role of multiple inflammatory markers and the INFLA score in the relationship between MetS and AA. Among the various chronic inflammation markers, we found that WBC had the highest mediating role, consistent with findings by Parikh et al., who highlighted the association between white blood cell differential counts and AA (8). The INFLA score, a marker of low-grade chronic inflammation, has been associated with numerous diseases (28, 29). Our study demonstrated that the INFLA score significantly mediated the association between MetS and its components with AA. Furthermore, MetS patients with high INFLA scores exhibited a substantially increased risk of AA, underscoring the potential role of chronic inflammation in AA pathogenesis. However, despite these findings, no significant interaction between MetS and INFLA scores was observed, suggesting that further studies are needed to elucidate this relationship.

Our study has several notable strengths. First, it is a large-scale prospective cohort study, offering detailed and comprehensive data on the development of MetS and its association with AA. Second, this is the first cohort study to investigate the relationship between MetS, inflammatory markers, and the risk of AA development, advancing our understanding of these interconnections. Furthermore, we emphasized the role and significance of the INFLA score and its related inflammatory markers, highlighting their potential as mediators in the MetS-AA relationship. These findings offer fresh insights and valuable guidance for the development of future prevention and intervention strategies targeting AA.

Our study has several limitations. First, as an observational study, it cannot establish a causal relationship between MetS, inflammatory markers, and the development of AA. Second, the UK Biobank does not provide detailed information on the size of aortic aneurysms, limiting our ability to include these data in our analysis. Third, we could not fully adjust for potential residual confounding factors, such as the influence of unmeasured comorbidities. Lastly, the predominantly white adult population in the UK Biobank may restrict the generalizability of our findings to other ethnic groups, necessitating further studies in more diverse populations.

In conclusion, we found that metabolic syndrome and its subcomponents were significantly associated with an increased risk of aortic aneurysm. Inflammation appears to play an important mediating role in this association. These findings underscore the importance of developing targeted preventive strategies, particularly for patients with MetS who exhibit higher levels of chronic inflammation.

## Data Availability

The datasets presented in this study can be found in online repositories. The names of the repository/repositories and accession number(s) can be found in the article/**Supplementary Material**.
