# Supplementary material for: Prospective analysis of metabolic syndrome and inflammation in aortic aneurysm risk: UK Biobank study
Source: Front Endocrinol (Lausanne). 2025 Jul 4;16:1612975. doi: 10.3389/fendo.2025.1612975 (PMC12270853; doi:10.3389/fendo.2025.1612975)
Supplement: Supplementary file 1 [file DataSheet1.docx]

**Supplementary Material**

Prospective Analysis of Metabolic Syndrome and Inflammation in Aortic Aneurysm Risk: UK Biobank Study

Xinyi Liu, Hao Liu, Chen Gong, Yipeng Ge, Haiou Hu, Zhiyu Qiao, Chengnan Li, Junming Zhu

**Supplementary method**

**Supplementary table 1.** Lifestyle assessment.

**Supplementary table 2.** Components of an ideal diet.

**Supplementary table 3.** Association of aortic aneurysm with metabolic syndrome components and INFLA.

**Supplementary table 4.** Sensitivity analysis of the association between metabolic syndrome and its components and aortic aneurysm.

**Supplementary table 5.** Subgroup Analysis of the Association Between Aortic Aneurysm Incidence, Metabolic Syndrome, and INFLA: Mediation and Interaction Analysis.

**Supplementary figure 1.** Flowchart of study participants.

**Supplementary figure 2.** Diagnostic plots for assessing post-propensity score matching data quality.

**Supplementary figure 3.** Mediation analysis of inflammatory markers in the association between metabolic syndrome and aortic aneurysm.

**Supplementary figure 4.** Mediation analysis of the INFLA score in the association between metabolic syndrome components and aortic aneurysm.

**Supplementary figure 5.** Nonlinear relationship between components of MetS and the incidence of aortic aneurysm.

**Supplementary figure 6.** Nonlinear relationship between INFLA components and aortic aneurysm incidence.

**Supplementary figure 7.** Evaluate the interaction between MetS and INFLA scores in relation to the incidence of aortic aneurysm.

Supplementary table 1. Lifestyle assessment.

| **Lifestyle factor** | **Field IDs** | **Lifestyle assessment** |
| --- | --- | --- |
| Smoking | 1239 1249 2644 | Past or current smoking  Smoked at least 100 cigarettes in their lifetime |
| Drinking | 1558 1568 1578 1588 1598 1608 5364 | More than two drinks per day for men More than one drink per day for women |
| Sleep pattern | 1160  1180 1200 1210 1220 | For each component of the sleep pattern, a score of 1 is assigned to high-risk components, while low-risk components are assigned a score of 0. The total sleep pattern score ranges from 0 to 5, with scores of 0–1 indicating a low-risk category, 2–3 representing a medium-risk category, and 4–5 corresponding to high-risk sleep patterns. |
| Physical activity | 971 981 991 1001 2624 2634 3637 3647 | The cohort was categorized into light, moderate, and heavy physical activity levels based on the tertile distribution of weekly physical activity Metabolic Equivalent of Task (MET) values. |
| Sedentary time | 1070  1080 | The cohort was divided into mild, moderate, and severe levels of sedentary behavior based on the tertile distribution of sedentary time. |
| Diet | Supplementary table 2 | A diet consisting of five or more key components is considered a healthy diet. |

Supplementary table 2. Components of an ideal diet.

| **Diet component** | **Field IDs** | **Intake goal** |
| --- | --- | --- |
| Fruit | 1309 1319 | ≥3 servings/day |
| Vegetable | 1289 1299 | ≥3 servings/day |
| Whole grains | 1438/1448 1458/1468 | ≥3 servings/day |
| Fish (Shell) | 1329 1339 | ≥2 servings/day |
| Dairy | 1408 1418 | ≥2 servings/day |
| Vegetable oils | 1428 1438 2654 | ≥2 servings/day |
| Refined grains | 1438/1448 1458/1468 | ≤2 servings/day |
| processed meats | 1349 3680 | ≤1 servings/day |
| Unprocessed meats | 1359 1369 1379 1389 3680 | ≤2 servings/day |
| Sugar-sweetened beverages | 6144 | No consumption |

Supplementary table 3. Association of aortic aneurysm with metabolic syndrome components and INFLA.

| **Characteristics** | **Unadjusted for INFLA score** | | **Adjusted for INFLA score** | |
| --- | --- | --- | --- | --- |
|  | **HR (95% CI)** | **P value** | **HR (95% CI)** | **P value** |
| Per component increment (Model1) | | | | |
| 0 | Reference |  | Reference |  |
| 1 | 1.50 (1.19-1.88) | <0.001 | 1.45 (1.16-1.82) | 0.004 |
| 2 | 2.07 (1.66-2.58) | <0.001 | 1.95 (1.56-2.43) | <0.001 |
| 3 | 2.50 (2.01-3.13) | <0.001 | 2.29 (1.84-2.88) | <0.001 |
| 4 | 3.33 (2.65-4.19) | <0.001 | 3.01 (2.39-3.79) | <0.001 |
| 5 | 3.33 (2.53-4.39) | <0.001 | 2.95 (2.24-3.90) | <0.001 |
| Per component increment (Model2) | | | | |
| 0 | Reference |  | Reference |  |
| 1 | 1.51 (1.20-1.89) | <0.001 | 1.47 (1.17-1.84) | <0.001 |
| 2 | 1.89 (1.51-2.37) | <0.001 | 1.81 (1.44-2.26) | <0.001 |
| 3 | 2.15 (1.71-2.73) | <0.001 | 2.04 (1.61-2.57) | <0.001 |
| 4 | 2.689 (2.09-3.45) | <0.001 | 2.52 (1.97-3.23) | <0.001 |
| 5 | 2.51 (1.86-3.39) | <0.001 | 2.33 (1.73-3.15) | <0.001 |
| Per component increment (Model3) | | | | |
| 0 | Reference |  | Reference |  |
| 1 | 1.51 (1.20-1.89) | <0.001 | 1.48 (1.18-1.85) | <0.001 |
| 2 | 1.85 (1.47-2.31) | <0.001 | 1.78 (1.42-2.23) | <0.001 |
| 3 | 2.05 (1.62-2.59) | <0.001 | 1.96 (1.55-2.48) | <0.001 |
| 4 | 2.49 (1.94-3.19) | <0.001 | 2.37 (1.85-3.05) | <0.001 |

Model 1, age and gender only; Model 2, further adjusting for education, self-reported ethnicity, Thomson deprivation index, employment, body mass index, and prevalent comorbidities (including history of cardiovascular diseases, chronic respiratory disease, chronic kidney disease, or chronic liver disease); Mode 3, with further adjustments to personal lifestyle, including diet, sleep patterns, sedentary time, and smoking and alcohol consumption. Abbreviations: INFLA score, Low-grade chronic inflammation score; HR, hazard ratio; CI, confidence interval.

Supplementary table 4. Sensitivity analysis of the association between metabolic syndrome and its components and aortic aneurysm.

| **Analyses** | **Incident Aortic Aneurysm** | |
| --- | --- | --- |
|  | **HR (95% CI)** | **P value** |
| **Simultaneous adjustment for inflammation test indicators** | | |
| Presence of MetS | 1.26 (1.14-1.38) | <0.001 |
| Per component increment | 1.152 (1.11-1.20) | <0.001 |
| 0 | Reference |  |
| 1 | 1.46 (1.16-1.83) | <0.001 |
| 2 | 1.74 (1.39-2.18) | <0.001 |
| 3 | 1.91 (1.51-2.41) | <0.001 |
| 4 | 2.30 (1.79-2.96) | <0.001 |
| 5 | 2.12 (1.57-2.86) | <0.001 |
| Hypertension | 1.25 (1.09-1.43) | <0.001 |
| Central obesity | 1.27 (1.14-1.42) | <0.001 |
| Hypertriglyceridemia | 1.12 (1.03-1.22) | <0.001 |
| Dyslipidemia | 1.37 (1.25-1.50) | <0.001 |
| Hyperglycemia | 0.93 (0.82-1.05) | 0.233 |
| **Weighted INFLA Scores** | | |
| Presence of MetS | 1.27 (1.16-1.40) | <0.001 |
| Per component increment | 1.16 (1.11-1.21) | <0.001 |
| 0 | Reference |  |
| 1 | 1.48 (1.18-1.85) | <0.001 |
| 2 | 1.78 (1.42-2.24) | <0.001 |
| 3 | 1.97 (1.56-2.49) | <0.001 |
| 4 | 2.38 (1.86-3.06) | <0.001 |
| 5 | 2.17 (1.61-2.94) | <0.001 |
| Hypertension | 1.26 (1.10-1.43) | <0.001 |
| Central obesity | 1.29 (1.16-1.45) | <0.001 |
| Hypertriglyceridemia | 1.13 (1.04-1.23) | 0.005 |
| Dyslipidemia | 1.38 (1.26-1.51) | <0.001 |
| Hyperglycemia | 0.93 (0.82-1.05) | 0.262 |
| **Excludes individuals with hypertension, diabetes, CVD, CLD, CKD, or CRD** | | |
| Presence of MetS | 1.33 (1.17-1.51) | <0.001 |
| Per component increment | 1.19 (1.13-1.26) | <0.001 |
| 0 | Reference |  |
| 1 | 1.37 (1.04-1.79) | 0.024 |
| 2 | 1.69 (1.28-2.22) | <0.001 |
| 3 | 1.84 (1.38-2.45) | <0.001 |
| 4 | 2.54 (1.86-3.46) | <0.001 |
| 5 | 2.09 (1.39-3.15) | <0.001 |
| Hypertension | 1.20 (1.02-1.41) | 0.021 |
| Central obesity | 1.28 (1.10-1.49) | 0.006 |
| Hypertriglyceridemia | 1.14 (1.02-1.27) | 0.022 |
| Dyslipidemia | 1.54 (1.37-1.72) | <0.001 |
| Hyperglycemia | 0.92 (0.77-1.11) | 0.432 |
| **Excluding events that occurred within the first three years of follow-up** | | |
| Presence of MetS | 1.28 (1.15-1.41) | <0.001 |
| Per component increment | 1.15 (1.11-1.21) | <0.001 |
| 0 | Reference |  |
| 1 | 1.47 (1.16-1.86) | <0.001 |
| 2 | 1.75 (1.39-2.21) | <0.001 |
| 3 | 1.95 (1.53-2.48) | <0.001 |
| 4 | 2.34 (1.81-3.03) | <0.001 |
| 5 | 2.09 (1.53-2.87) | <0.001 |
| Hypertension | 1.27 (1.11-1.45) | <0.001 |
| Central obesity | 1.30 (1.16-1.47) | <0.001 |
| Hypertriglyceridemia | 1.14 (1.04-1.24) | 0.002 |
| Dyslipidemia | 1.34 (1.22-1.47) | <0.001 |
| Hyperglycemia | 0.92 (0.81-1.06) | 0.255 |
| **Multiple imputation** | | |
| Presence of MetS | 1.27 (1.16-1.40) | <0.001 |
| Per component increment | 1.16 (1.11-1.20) | <0.001 |
| 0 | Reference |  |
| 1 | 1.47 (1.17-1.84) | <0.001 |
| 2 | 1.76 (1.41-2.21) | <0.001 |
| 3 | 1.94 (1.54-2.45) | <0.001 |
| 4 | 2.34 (1.82-2.99) | <0.001 |
| 5 | 2.14 (1.58-2.88) | <0.001 |
| Hypertension | 1.25 (1.10-1.43) | <0.001 |
| Central obesity | 1.29 (1.15-1.44) | <0.001 |
| Hypertriglyceridemia | 1.13 (1.04-1.23) | 0.004 |
| Dyslipidemia | 1.37 (1.25-1.49) | <0.001 |
| Hyperglycemia | 0.93 (0.82-1.05) | 0.252 |
| **Data after 1:1 propensity score matching** | | |
| Presence of MetS | 1.24 (1.12-1.36) | <0.001 |
| Per component increment | 1.12 (1.07-1.17) | <0.001 |
| 0 | Reference |  |
| 1 | 1.32 (1.05-1.65) | 0.018 |
| 2 | 1.40 (1.11-1.75) | 0.003 |
| 3 | 1.62 (1.28-1.75) | <0.001 |
| 4 | 1.77 (1.38-2.27) | <0.001 |
| 5 | 1.89 (1.40-2.55) | <0.001 |
| Hypertension | 1.16 (1.02-1.32) | 0.028 |
| Central obesity | 1.17 (1.04-1.30) | 0.006 |
| Hypertriglyceridemia | 1.10 (1.01-1.19) | 0.031 |
| Dyslipidemia | 1.29 (1.18-1.41) | <0.001 |
| Hyperglycemia | 0.97 (0.86-1.09) | 0.622 |

All analyses were adjusted for age, sex, education, self-reported ethnicity, Thomson deprivation index, employment, body mass index, and prevalent comorbidities (including history of cardiovascular disease, chronic respiratory disease, chronic kidney disease, or chronic liver disease) and lifestyle (including diet, physical activity, sleep patterns, sedentary time, and smoking and alcohol consumption). Abbreviations: CI, confidence interval; HR, hazard ratio; MetS: Metabolic Syndrome; INFLA score, Low-grade chronic inflammation score; CVD, cardiovascular disease; CLD, Chronic liver disease; CKD, Chronic kidney disease; CRD, Chronic respiratory diseases.

Supplementary table 5. Subgroup Analysis of the Association Between Aortic Aneurysm Incidence, Metabolic Syndrome, and INFLA: Mediation and Interaction Analysis.

| **Characteristics** | **No of cases /person-years** | **HR (95% CI)** | **P value** | **Mediation proportion (%,95%CI)**† |
| --- | --- | --- | --- | --- |
| **Sex** |  |  |  |  |
| Male | 1903/147087 | 1.29 (1.16-1.43) | <0.001 | 6.3 (2.6-11.2) |
| Female | 479/165418 | 1.20 (0.96-1.49) | 0.112 | 9.9 (1.5-19.4) |
| P for interaction* |  |  | 0.430 |  |
| **Age** |  |  |  |  |
| ≥60 years | 1766/134046 | 1.35 (1.21-1.51) | <0.001 | 5.9 (3.2-11.1) |
| <60 years | 616/178459 | 1.31 (1.08-1.59） | <0.01 | 5.6 (0.7-18.9) |
| P for interaction |  |  | 0.672 |  |
| **BMI** |  |  |  |  |
| Normal BMI | 507/165418 | 1.06 (0.82-1.37) | 0.663 | 4.2 (1.2-12.3) |
| Abnormal BMI | 1875/210611 | 1.30 (1.18-1.43) | <0.001 | 7.3 (3.4-16.1) |
| P for interaction |  |  | 0.252 |  |
| **Smoking** |  |  |  |  |
| No | 738/171780 | 1.06 (0.89-1.27) | 0.483 | 3.3 (1.9-11.6) |
| Yes | 1644/140725 | 1.37 (1.22-1.54) | <0.001 | 6.6 (4.2-12.8) |
| P for interaction |  |  | 0.016 |  |
| **Hypertension** |  |  |  |  |
| No | 286/91251 | 1.44 (0.95-2.22) | 0.101 | 5.4 (1.4-15.6) |
| Yes | 2096/218872 | 1.28 (1.16-1.42) | <0.001 | 7.5 (3.1-17.2) |
| P for interaction |  |  | 0.435 |  |

†The mediation proportion refers to the extent to which the INFLA score mediates the relationship between metabolic syndrome and the occurrence of aortic aneurysms in each subgroup.

*The P for interaction indicates whether the difference in risk ratios between two subgroups—individuals without metabolic syndrome and an INFLA score of -16 to 0, compared to those with metabolic syndrome and an INFLA score of 6 to 16—is statistically significant or not.

Abbreviations: INFLA score, Low-grade chronic inflammation score; HR, hazard ratio; CI, confidence interval.

Supplementary figure 1. Flowchart of study participants.


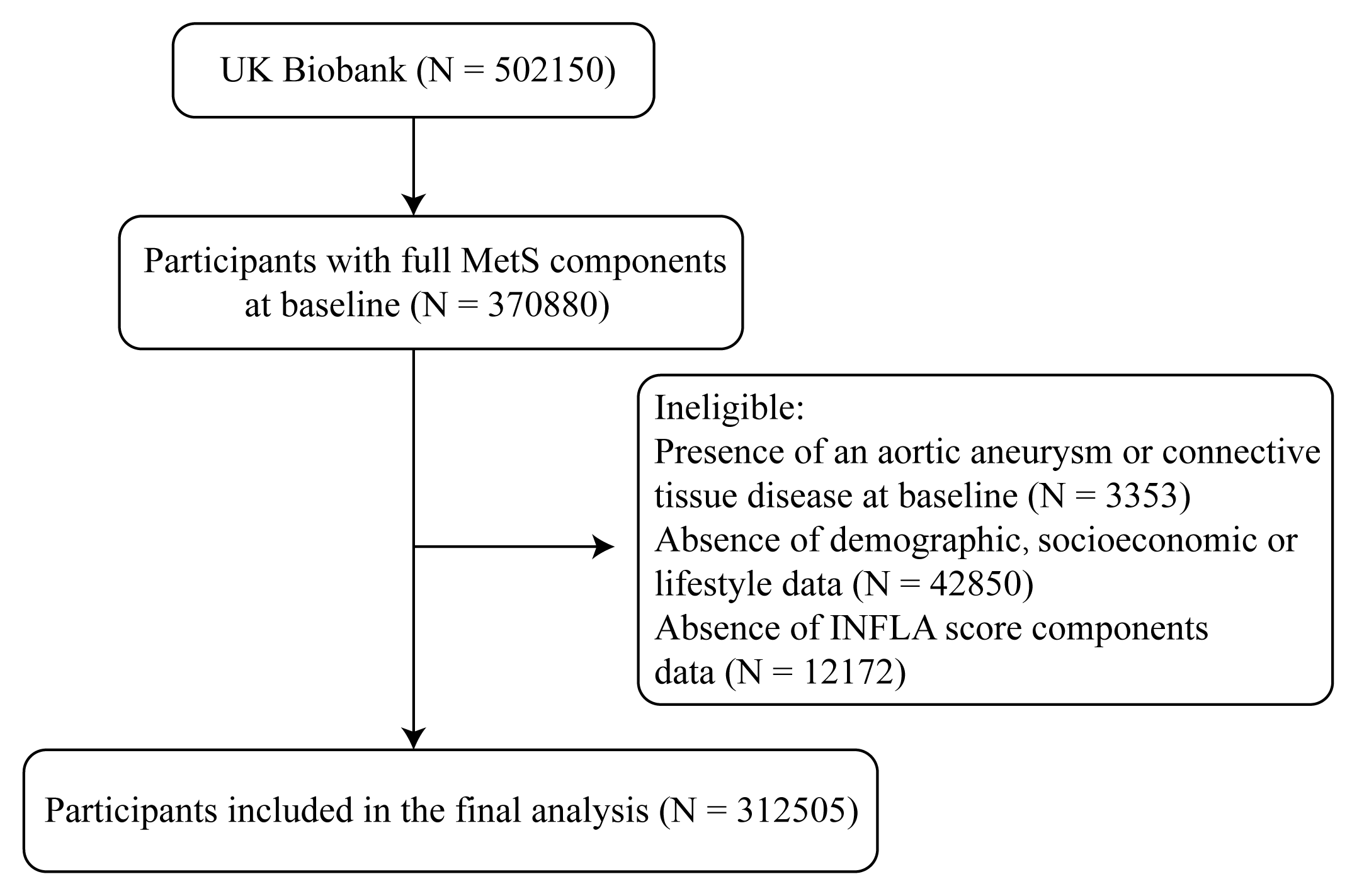


Supplementary figure 2. Diagnostic plots for assessing post-propensity score matching data quality.


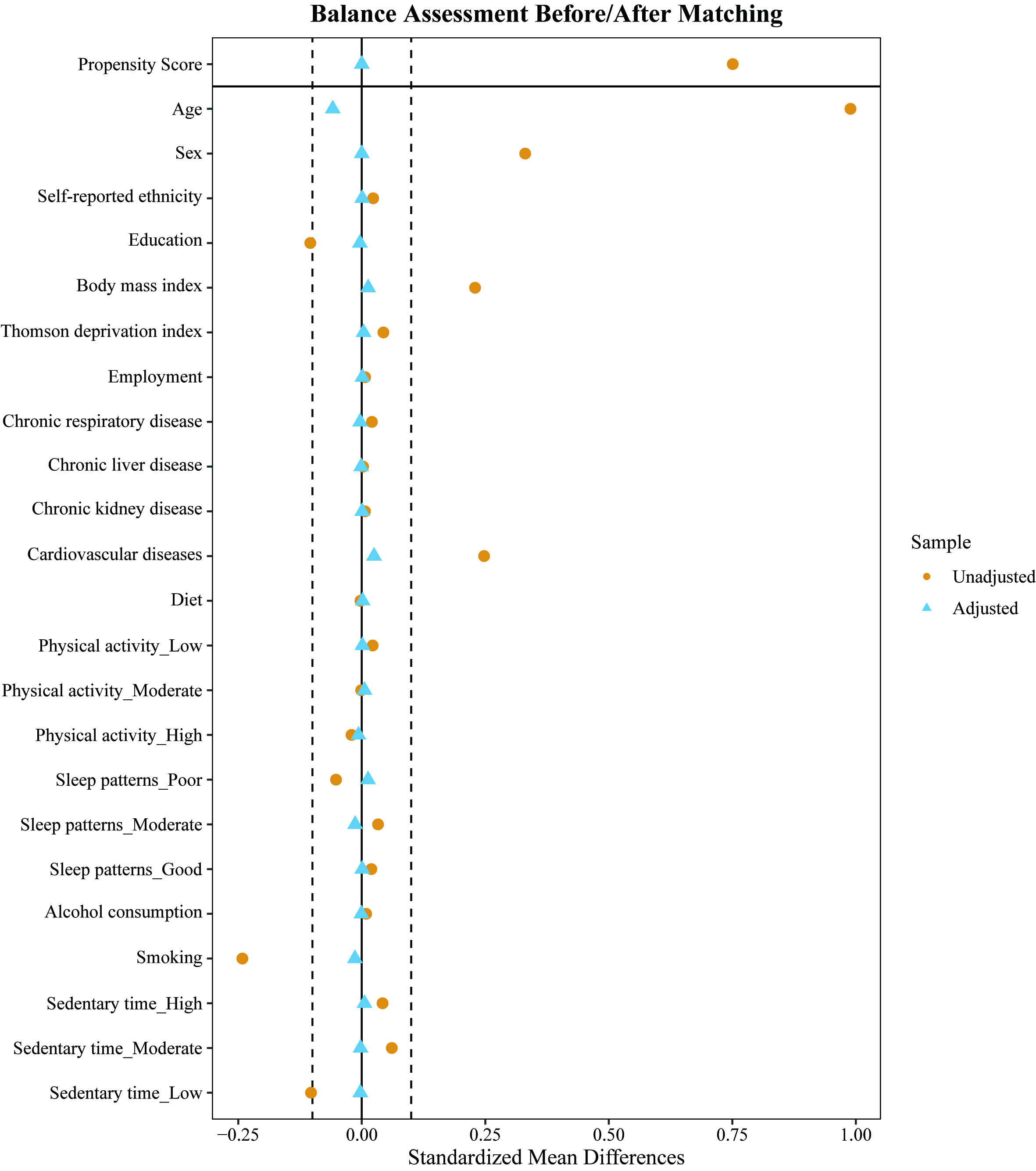


Supplementary figure 3. Mediation analysis of inflammatory markers in the association between metabolic syndrome and aortic aneurysm.


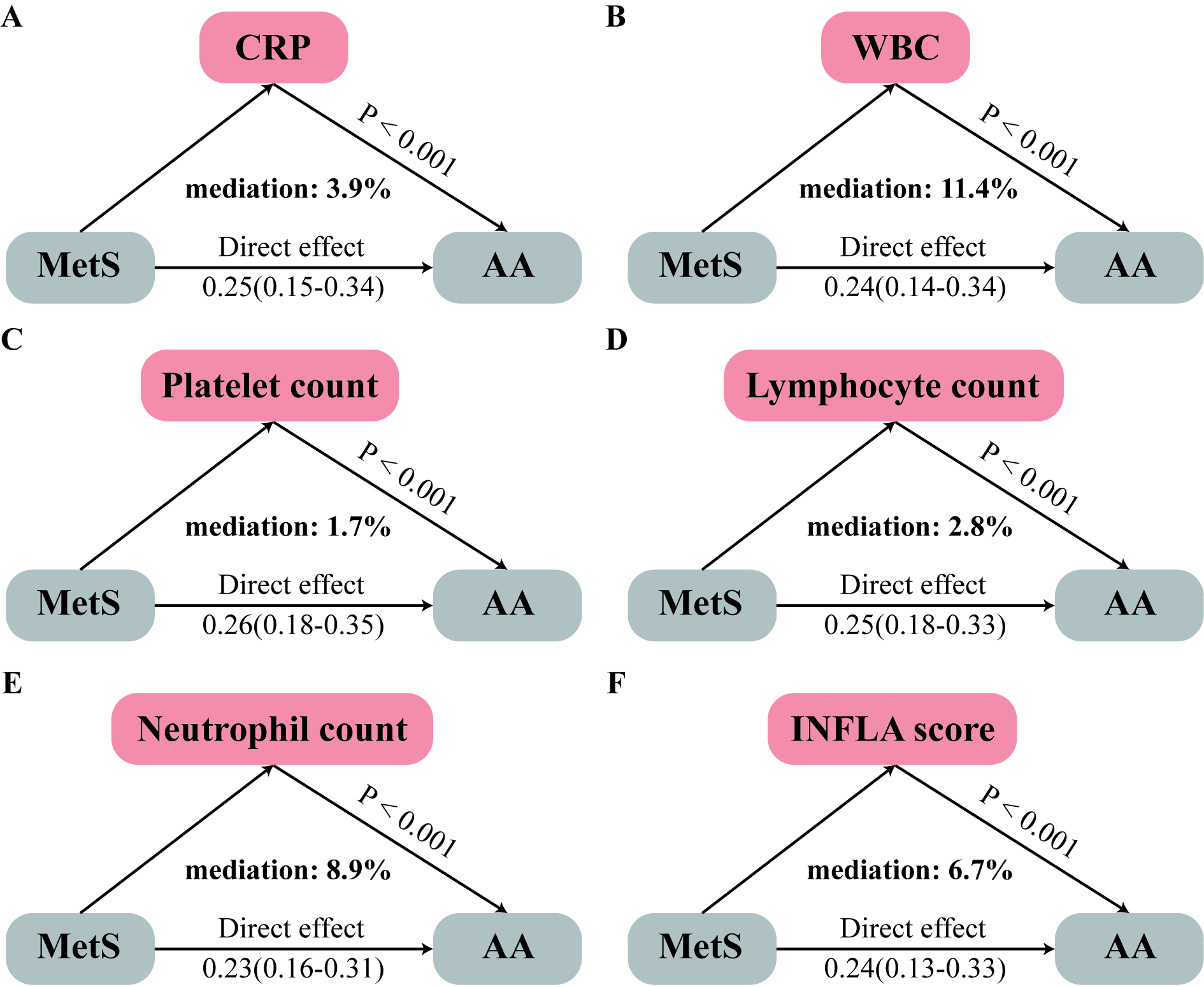


(A-F). All analyses were adjusted for age, sex, education, self-reported ethnicity, Thomson deprivation index, employment, body mass index, and prevalent comorbidities (including history of cardiovascular disease, chronic respiratory disease, chronic kidney disease, or chronic liver disease) and lifestyle (including diet, physical activity, sleep patterns, sedentary time, and smoking and alcohol consumption).

Abbreviations: MetS, Metabolic Syndrome; AA, Aortic aneurysm; CRP, C-reaction protein; WBC, white blood cell; INFLA score, Low-grade chronic inflammation score;

Supplementary figure 4. Mediation analysis of the INFLA score in the association between metabolic syndrome components and aortic aneurysm.


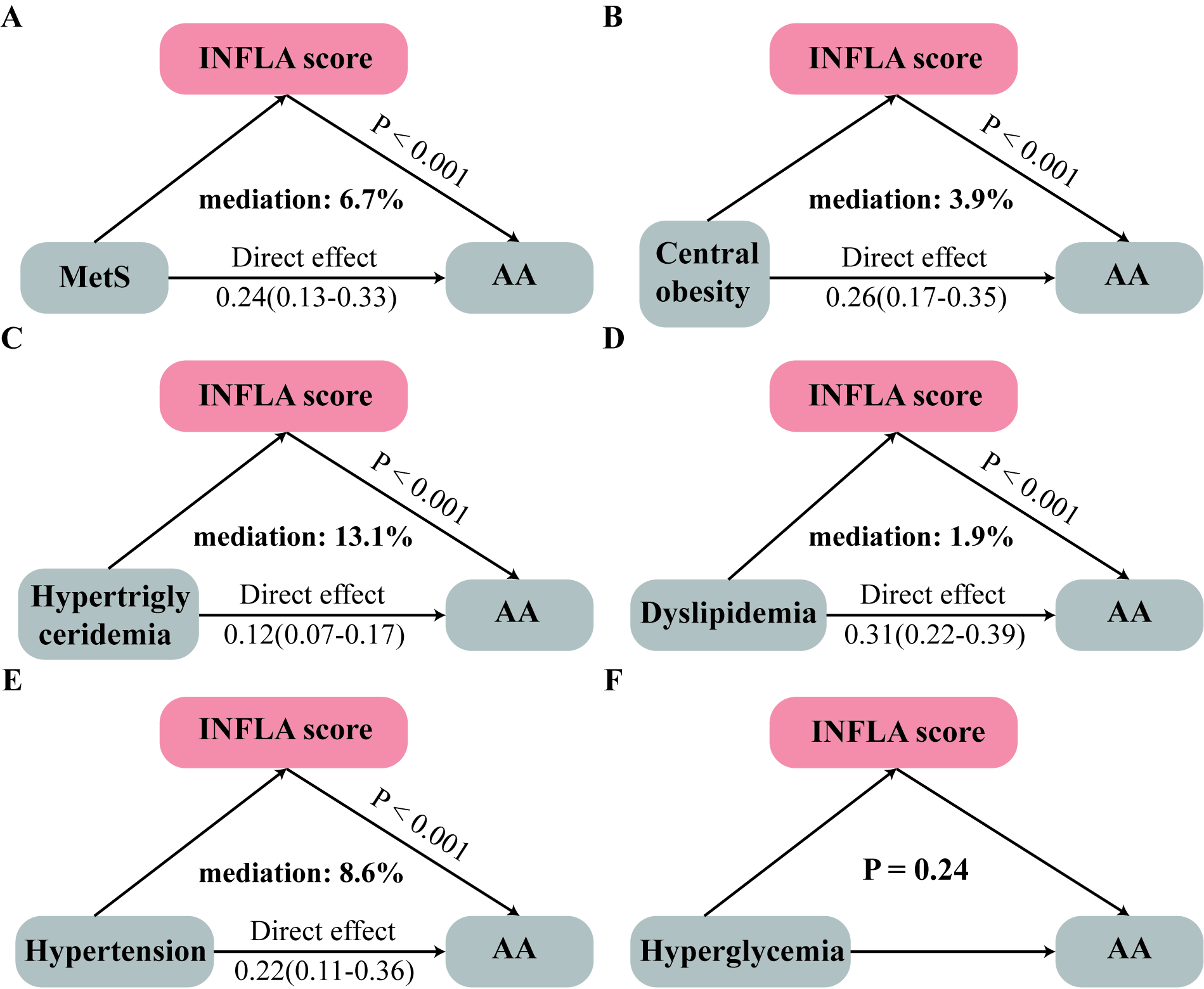


(A-F). All analyses were adjusted for age, sex, education, self-reported ethnicity, Thomson deprivation index, employment, body mass index, and prevalent comorbidities (including history of cardiovascular disease, chronic respiratory disease, chronic kidney disease, or chronic liver disease) and lifestyle (including diet, physical activity, sleep patterns, sedentary time, and smoking and alcohol consumption).

Abbreviations: MetS, Metabolic Syndrome; AA, Aortic aneurysm; INFLA score, Low-grade chronic inflammation score.

Supplementary figure 5. Nonlinear relationship between components of MetS and the incidence of aortic aneurysm.


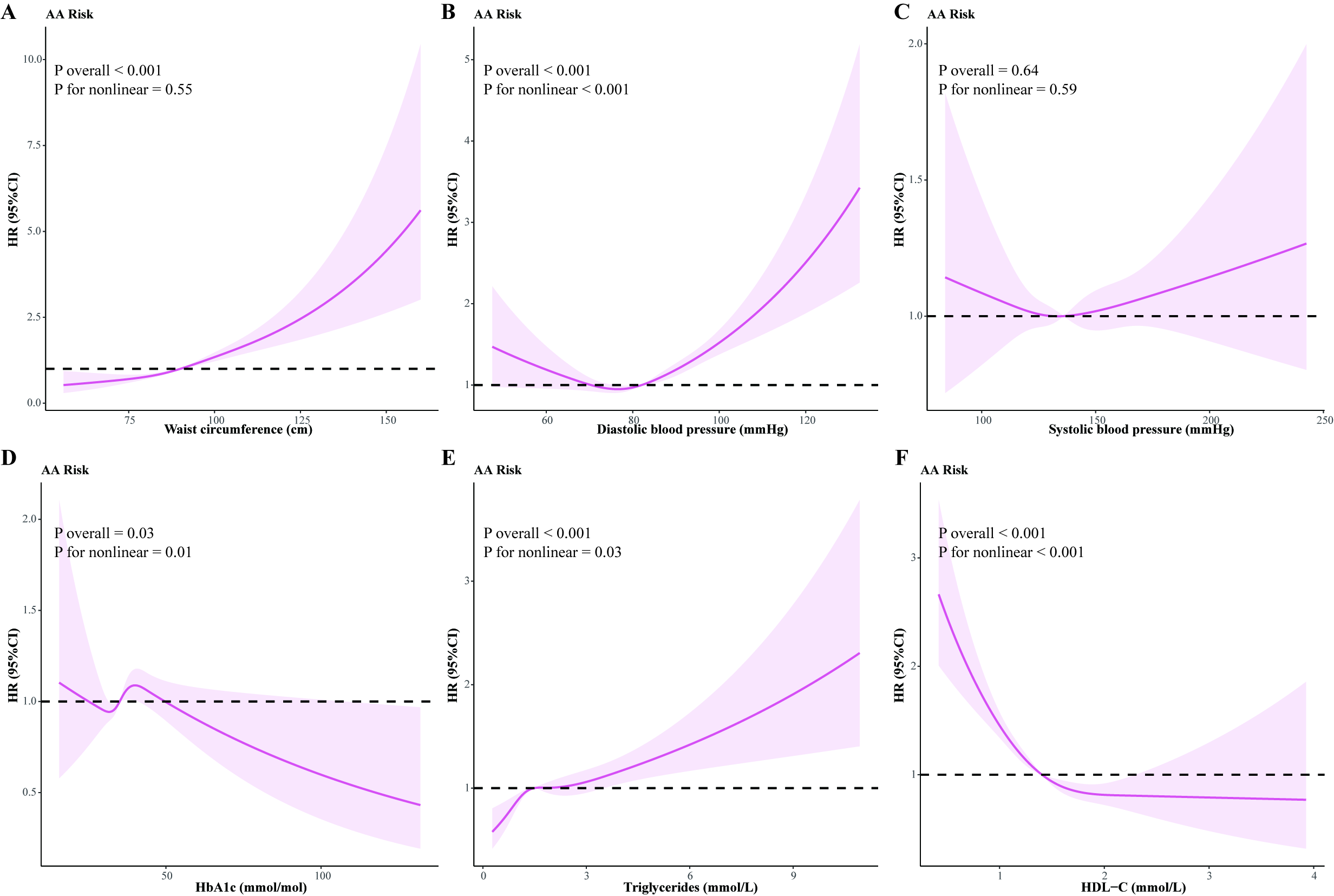


All analyses were adjusted for age, sex, education, self-reported ethnicity, Thomson deprivation index, employment, body mass index, and prevalent comorbidities (including history of cardiovascular disease, chronic respiratory disease, chronic kidney disease, or chronic liver disease) and lifestyle (including diet, physical activity, sleep patterns, sedentary time, and smoking and alcohol consumption).

Abbreviations: CI, confidence interval; HR, hazard ratio; MetS, Metabolic Syndrome; HDL-C, High-density lipoprotein cholesterol.

Supplementary figure 6. Nonlinear relationship between INFLA components and aortic aneurysm incidence.


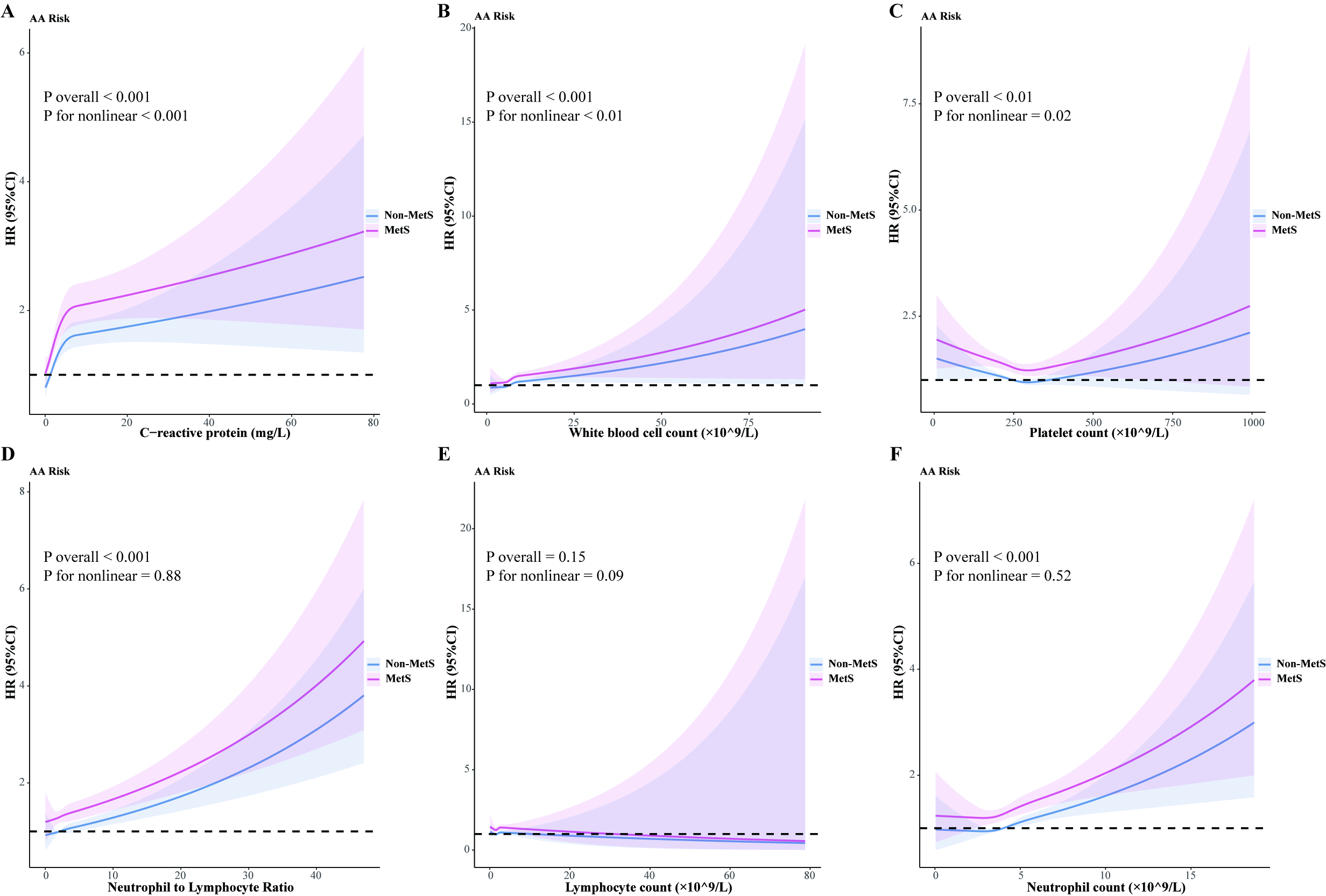


All analyses were adjusted for age, sex, education, self-reported ethnicity, Thomson deprivation index, employment, body mass index, and prevalent comorbidities (including history of cardiovascular disease, chronic respiratory disease, chronic kidney disease, or chronic liver disease) and lifestyle (including diet, physical activity, sleep patterns, sedentary time, and smoking and alcohol consumption).

Abbreviations: CI, confidence interval; HR, hazard ratio; INFLA score, Low-grade chronic inflammation score.

Supplementary figure 7. Evaluate the interaction between MetS and INFLA scores in relation to the incidence of aortic aneurysm.


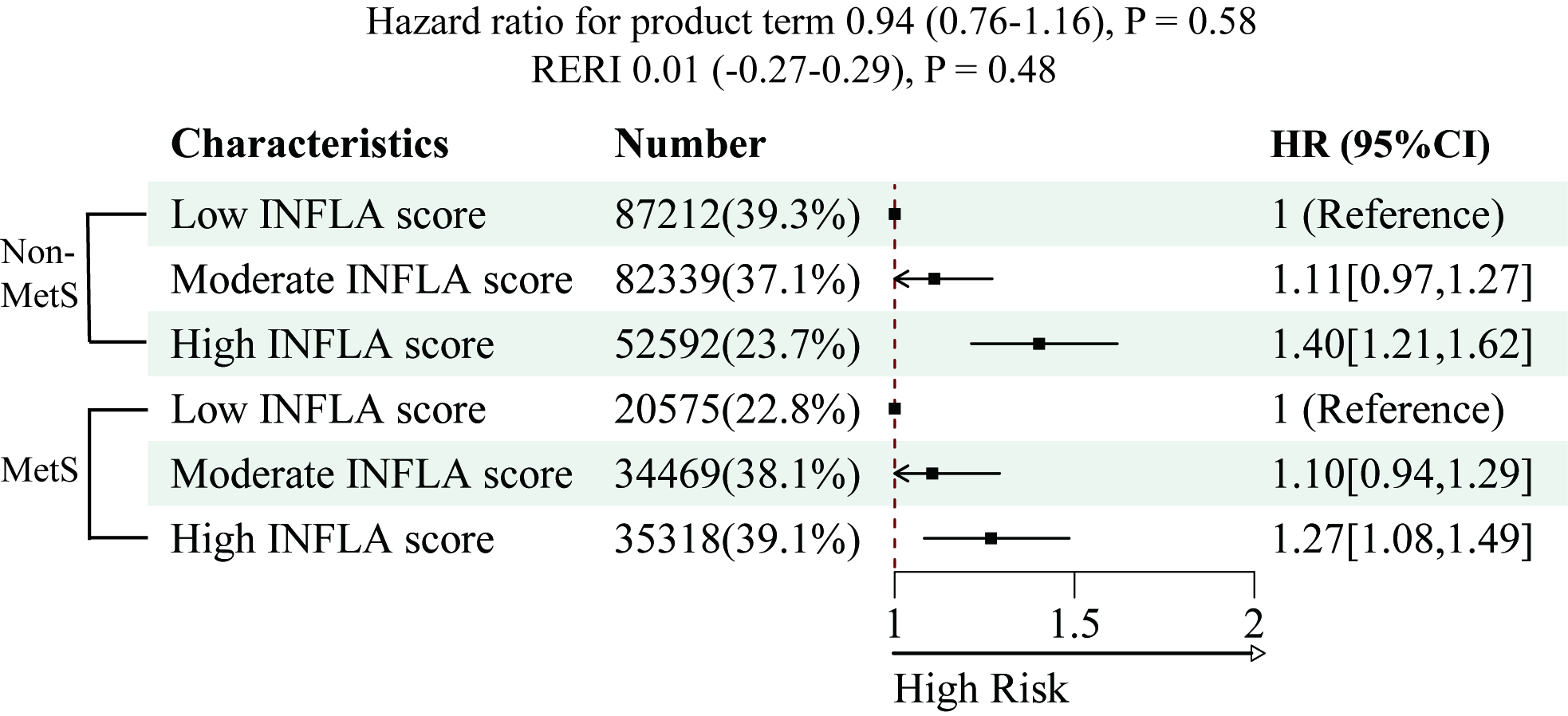


The analysis adjusted for age, sex, education, self-reported ethnicity, Thomson deprivation index, employment, body mass index, and prevalent comorbidities (including history of cardiovascular disease, chronic respiratory disease, chronic kidney disease, or chronic liver disease) and lifestyle (including diet, physical activity, sleep patterns, sedentary time, and smoking and alcohol consumption).

Multiplicative interactions were evaluated using hazard ratios for the association between unhealthy lifestyle and shift work, with statistical significance determined when the confidence intervals excluded 1. Additive interactions were assessed using the relative excess risk due to interaction (RERI), with statistical significance determined when the confidence intervals excluded 0.

Abbreviations: CI, confidence interval; HR, hazard ratio; INFLA score, Low-grade chronic inflammation score; MetS, Metabolic Syndrome; RERI, Relative excess risk due to interaction.
